# Escalation strategies for combination therapy Phase I trials

**DOI:** 10.1002/pst.1497

**Published:** 2012-03-12

**Authors:** Michael J Sweeting, Adrian P Mander

**Affiliations:** *MRC Biostatistics Unit Institute of Public Health Robinson Way, Cambridge Cambridgeshire UK.

**Keywords:** dose-escalation, Bayesian adaptive designs, optimal design, combination therapy, Phase I trials, Bayesian D-optimality

## Abstract

Phase I clinical trials aim to identify a maximum tolerated dose (MTD), the highest possible dose that does not cause an unacceptable amount of toxicity in the patients. In trials of combination therapies, however, many different dose combinations may have a similar probability of causing a dose-limiting toxicity, and hence, a number of MTDs may exist. Furthermore, escalation strategies in combination trials are more complex, with possible escalation/de-escalation of either or both drugs. This paper investigates the properties of two existing proposed Bayesian adaptive models for combination therapy dose-escalation when a number of different escalation strategies are applied. We assess operating characteristics through a series of simulation studies and show that strategies that only allow ‘non-diagonal’ moves in the escalation process (that is, both drugs cannot increase simultaneously) are inefficient and identify fewer MTDs for Phase II comparisons. Such strategies tend to escalate a single agent first while keeping the other agent fixed, which can be a severe restriction when exploring dose surfaces using a limited sample size. Meanwhile, escalation designs based on Bayesian D-optimality allow more varied experimentation around the dose space and, consequently, are better at identifying more MTDs. We argue that for Phase I combination trials it is sensible to take forward a number of identified MTDs for Phase II experimentation so that their efficacy can be directly compared. Researchers, therefore, need to carefully consider the escalation strategy and model that best allows the identification of these MTDs. Copyright © 2012 John Wiley & Sons, Ltd.

## 1 INTRODUCTION

In recent years, combination therapy treatments have become more widespread, giving rise to a number of proposed models for estimating the maximum tolerated dose (MTD) in Phase I combination trials [1–5]. The aims and designs of two-agent Phase I trials are more complex than that of a single-agent trial, and a multitude of MTDs with a similar toxicity profile can be identified. Indeed, a number of drug combinations could be recommended for Phase II (RPII) experimentation so as to directly compare their efficacy. In theory, in a two-dimensional setting, an infinite number of possible dose combinations will achieve the same target toxicity level (TTL), assuming a continuous dose-toxicity surface. In practice, however, such choices are often restricted by predefining a set of dose combinations used for experimentation.

Combination drug Phase I trials often have rich prior information available on the dose-toxicity response from single-agent studies, which should be utilised to make the trial more efficient. For this reason, a number of authors have suggested using Bayesian parametric models to describe the dose-toxicity surface, where model parameters can be separated into those that relate to the marginal dose-toxicity response and those that relate to the ‘interaction’ between the two agents [2–4]. In particular, the three-parameter copula-type regression model proposed by Yin and Yuan 2009 is an extension of the popular continuous reassessment method (CRM) 1990 used in single-agent dose-finding trials, whereas the six-parameter model, proposed by Thall *et al.* 2003, is simplified into a logistic model, marginally, over each single agent.

Less attention, however, has been given to the performance of such models under different escalation strategies, when only a discrete set of dose combinations are available for experimentation. Different escalation strategies may result in different MTDs being identified and recommended for Phase II evaluation 2010. In single-agent trials, the risk of overdosing can be avoided by dose-escalation strategies that do not skip any predefined dose levels. However, in combination therapy trials, the set of admissible doses, with which the next cohort can be treated, is more complex. A number of authors have suggested escalation to neighbouring dose combinations within the two-dimensional space, with both agents increased concurrently 2003 and where only one agent is increased at a time [2,4].

In this paper, we consider different strategies for escalation and for the search of the dose-toxicity space, and show how these strategies affect the number and suitability of the RPII doses. We compare diagonal escalation where both drugs are increased simultaneously with escalation where only one drug is increased at a time between patient cohorts. We show how efficiency, gained through allowing ‘positive’ diagonal escalation, must be traded-off against an increased risk of overdosing. In combination therapies, however, the severity of overdosing may be less pronounced because dose ranges are likely to be more targeted, which is due to previous Phase I experimentation of single agents. In addition, we also propose strategies in moving between dose combinations based on either selecting the next dose whose estimated probability of dose-limiting toxicity (DLT) is closest to the TTL or using a Bayesian D-optimality criteria. The latter allows the design to search the dose-toxicity space more fully and to identify a larger number of MTDs. We propose that RPII doses be chosen on the basis of the estimated probability of DLT being within a tolerance interval of the TTL to allow more than one combination to be recommended for future evaluation, and we restrict our choice of RPII doses to only combinations that have been experimented on within the course of the trial. From this definition, we contrast two proposed models in their ability to identify the RPII dose combinations by using a set of simulation studies.

## 2 PARAMETRIC MODELS FOR COMBINATION THERAPY PHASE I TRIALS

We shall start by reviewing two proposed parametric models used in combination therapy Phase I trials: a six-parameter model 2003 and a three-parameter copula-type model 2009.

The model proposed by Thall *et al.* 2003 uses four parameters to describe the probability of toxicity at the margins (i.e. when each drug is used in isolation) and two parameters to describe the magnitude and shape of the dose-toxicity curve when the drugs are used in combination. Specifically, let ***x*** = (*x*_*Ai*_,*x*_*Bj*_) denote the dose combination when drug A is used at level *i* (*i* = 1, …, *I*) and drug B is used at level *j* (*j* = 1, …, *J*). The probability of a DLT is then given by

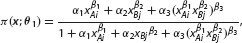
(1)
where the six parameters ***θ***_1_ = (*α*_1_,*β*_1_,*α*_2_,*β*_2_,*α*_3_,*β*_3_) are all positive. Suitable priors can be obtained for the parameters *α*_1_,*α*_2_,*β*_1_ and *β*_2_ by using data from single-agent trials or eliciting opinions from physicians. For the interaction parameters, *α*_3_ and *β*_3_, Thall *et al.* recommend using reasonably vague Gamma priors.

Yin and Yuan 2009 propose a copula-type model with three parameters, ***θ***_2_ = (*Δ*,*ψ*,*γ*). The model is of the following form:

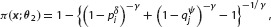
(2)
where *p*_*i*_ is a prespecified ‘best guess’ probability of DLT when drug A is used in isolation at level *i*, and *q*_*j*_ a ‘best guess’ probability of DLT when drug B is used in isolation at level *j*. These quantities are fixed in advance using prior knowledge (and are sometimes referred to as the ‘skeleton’ in a CRM). A monotonically increasing sequence is specified for both the *p*s and the *q*s. Nevertheless, in order to reflect the fact that the marginal probabilities are uncertain, the true probabilities are taken as 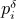 and 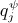, where *δ* and *ψ* are unknown parameters with a prior mean equal to one. The final model parameter is *γ* > 0, which characterises any interaction between the drugs. This copula model has been regarded as a multivariate extension of the CRM power model because if drug A is used in isolation, then *q* = 0, and hence, 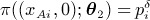. Similarly, when drug B is used in isolation, 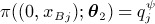.

## 3 DECISION RULES FOR ESCALATION

Suppose that *n* patients have currently been treated in the trial. The data can be summarised by the doses each patient received and the toxicity outcome indicators (*Y* = 1 for a DLT, and *Y* = 0 otherwise); hence, ***Z***_*n*_ = {(***x***_*k*_,*Y*_*k*_),*k* = 1, …, *n*}. Let *f*(***θ***) denote the prior distribution for the parameter vector ***θ***, where ***θ*** = ***θ***_1_ for the six-parameter model and ***θ*** = ***θ***_2_ for the three-parameter model. The posterior distribution after *n* patients by Bayes theorem is

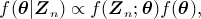

where the likelihood is binomial and given by

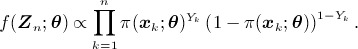


The choice of dose combination for patient (*n* + 1) is based upon the posterior distribution. For safety purposes and for the prevention of ‘dose skipping’, the set of doses for patient (*n* + 1) may be restricted to dose combinations close to the current combination, ***x***_*n*_. These sets of doses will be labelled the *admissible* doses for patient (*n* + 1). A number of possible approaches in choosing the admissible dose set are described in Section 4. For now, suppose that Ω represents our chosen set of admissible doses. One approach in choosing the next dose combination from this admissible set is to find the dose combination whose posterior mean toxicity is closest to the TTL *ν*, that is

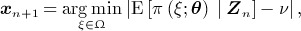
(3)
where 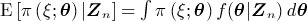. In a decision theoretic framework, this is equivalent to minimising the posterior expected loss with respect to the loss function

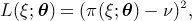

for *ξ* ∈ Ω 1967. This approach is commonly used in many single-agent dose-finding designs [4,6,9] and can be readily adapted to the combination therapy setting. We shall subsequently refer to an escalation strategy based on Equation (3) as decision rule *D*1.

An alternative approach is to select the next dose on the basis of a constrained Bayesian D-optimality design [3,10]. For example, within a two-dimensional dose-finding trial, it may be the case that among the admissible dose sets for patient (*n* + 1), a number of dose combinations have an estimated probability of toxicity within a small tolerance of the TTL. The dose whose posterior mean probability of DLT is closest to the TTL could be chosen, as described previously, or the investigator may wish to select the next dose on the basis of maximising the information of the model parameters while still assigning a dose that is close to the TTL. Let 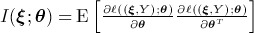
denote the Fisher information matrix associated with treating a patient at dose combination ***ξ***, given the parameters ***θ***, and where *ℓ* ((***ξ***,*Y*);***θ***) = log *f* ((***ξ***,*Y*);***θ***) is the log likelihood function. Bayesian D-optimality assigns patient *n* + 1 to dose ***x***_*n* + 1_ on the basis of maximising the posterior expectation of the log determinant of the information matrix given the current data, that is,

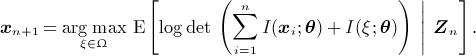
(4)

In order to ensure that patients are not assigned to highly toxic doses with this design, the admissible dose set Ω should be further restricted to doses that have toxicity within a certain tolerance of the TTL. We achieve this by restricting the admissible set, after *n* patients have been recruited, to those doses whose posterior mean probability of DLT is within a tolerance *ϵ* of the TTL, that is

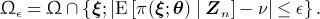
(5)

An alternative restricted dose set includes dose combinations that, with some large degree of probability, have a probability of DLT less than a maximal acceptable toxicity 2003. If the restricted dose set is empty, that is there are no admissible doses whose posterior mean probability of DLT is within *ϵ* of the TTL, then the next dose combination is chosen as the one whose posterior mean probability of DLT is closest to the TTL, as in Equation (3), subject to the original admissibility constraints Ω. An escalation strategy, based on D-optimality equation (4) and restriction (5), will be labelled strategy *D*2.

## 4 ADMISSIBLE DOSES

**Figure 1:**
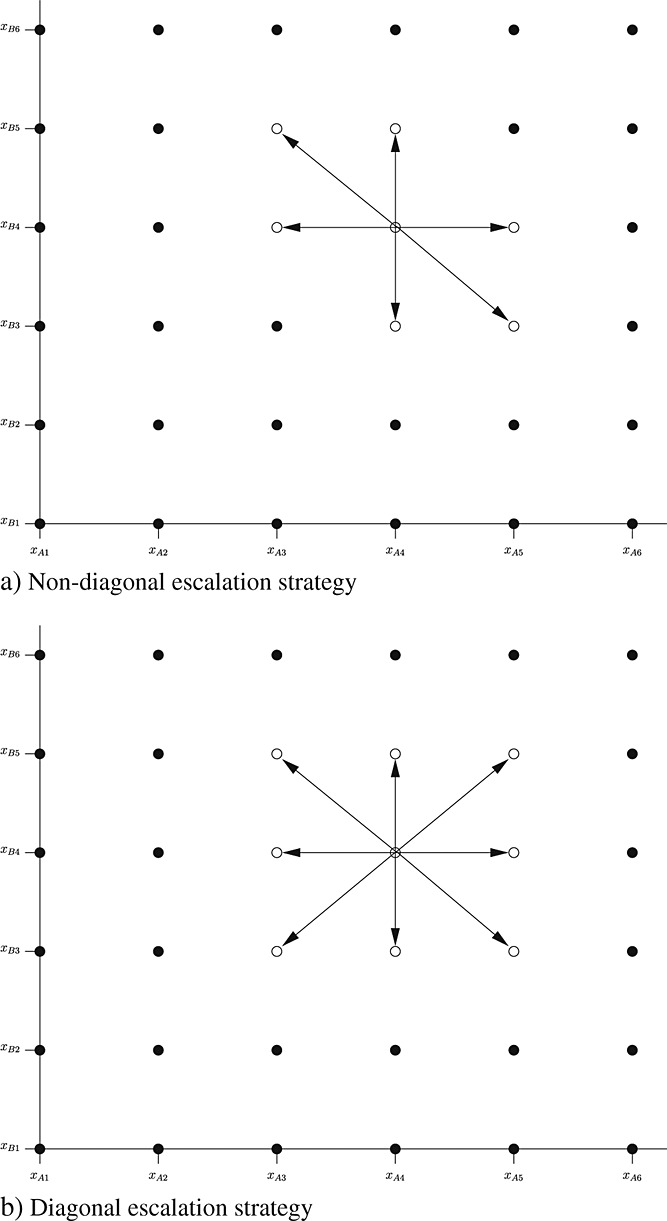
Admissible dose-escalation strategies using neighbouring dose combinations.

Overdosing is a major concern in Phase I trials, and in order to address this problem, designs often incorporate additional constraints to prevent skipping of predefined dose levels. In a combination therapy trial, this is equivalent to only allowing the next cohort to be treated at one dose level above the current prescribed dose for drug A, whereas drug B remains fixed, or one dose level above the current level for drug B, whereas drug A is kept fixed. The set of admissible dose combinations for the next cohort are therefore restricted to the neighbouring dose combinations in the two-dimensional space, without allowing diagonal moves (Figure 1(a)), as recommended by Yin and Yuan 2009. Such an admissible dose set will be labelled Ω_1_.

A slightly less conservative approach to dose finding is to additionally allow diagonal escalation, whereby both drugs are increased simultaneously (Figure 1(b)). This is equivalent to skipping one dose combination in the non-diagonal design because it would take two steps to reach this dose combination using such a design. However, in the context of combination therapy, such escalations may be tolerable (at least for the clinical trialist) because both drugs are likely to already have a known toxicity profile from earlier Phase I experimentation. Hence, the dose ranges used in the combination trial are likely to be more targeted. An admissible dose set based on diagonal escalation is labelled Ω_2_.

**Figure 2:**
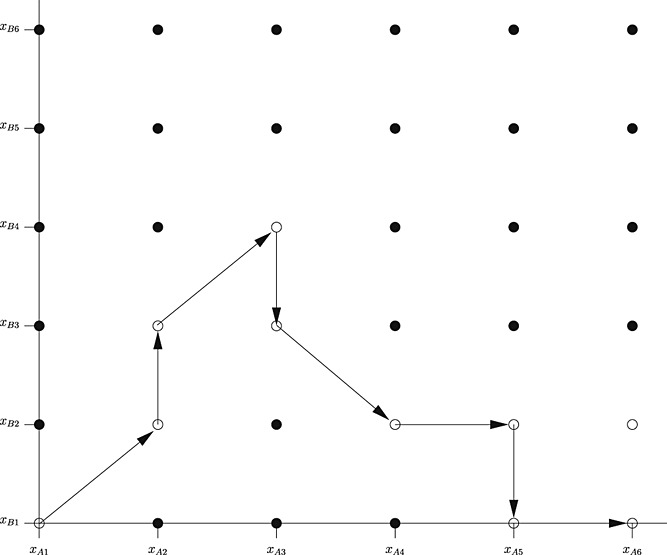
Admissible dose-escalation strategy using neighbouring and previously experimented dose combinations.

A third approach is to allow diagonal escalation to neighbouring combinations while also allowing the administration of any previously experimented dose combination. This has the advantage of allowing the design to explore more efficiently around the dose space. For example, suppose that *n* cohorts have been treated thus far in the trial and escalation has proceeded as depicted in Figure 2, using any neighbouring dose combinations as admissible doses. The current cohort are treated at combination (*x*_*A*6_,*x*_*B*1_). The majority of cohorts have been treated at high levels of drug A and low levels of drug B. However, this current imbalance in the allocation of doses means that little has been learned about the toxicity when low doses of drug A and high doses of drug B are given. Designs based on D-optimality are likely to propose escalation to such dose combinations, but a jump from the current dose combination to, say, dose combination (*x*_*A*1_,*x*_*B*6_) would involve recruiting five more cohorts if only neighbouring dose combinations are admissible. Allowing jumps to previously experimented dose combinations would, in this example, allow a jump directly to combination (*x*_*A*3_,*x*_*B*4_), with the hope of making the overall design more efficient. An admissible dose set based on neighbouring and previously experimented doses is labelled Ω_3_.

## 5 SIMULATIONS

We investigate the operating characteristics of both decision rules *D*1 and *D*2, described in Section 3, by using the admissible dose sets Ω_1_, Ω_2_ and Ω_3_, described in Section 4. Note that, for the D-optimal design (*D*2), each admissible dose set is further restricted by Equation (5).

**Figure 3:**
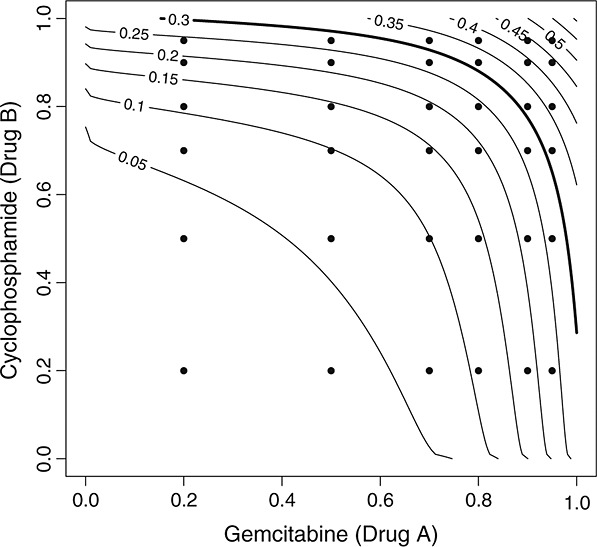
Contour plot of prior mean toxicity for Gemcitabine (Drug A) + Cyclophosphamide (Drug B). The bold line shows dose combinations at the target toxicity level (TTL) of 30%.

Simulations are conducted for both the six-parameter and three-parameter models, described in Section 2. The operating characteristics of the approaches are illustrated using a combination therapy cancer trial of Gemcitabine and Cyclophosphamide, which is described fully in Thall *et al.* [Bibr b3]. Priors for the six-parameter model, shown in Table 1, were elicited from expert physicians, and these give rise to a toxicity surface, as shown in Figure 3, where the prior mean probability of DLT contours are plotted. We consider six dose levels which can be used for each drug. These are standardised to their known MTD doses when each drug is used in isolation. Hence, dose combination (1,0) refers to the single-agent MTD for drug A, and (0,1) refers to the single-agent MTD for drug B. The six dose levels are as follows: (0.2,0.5,0.7,0.8,0.9,0.95). These were chosen for the experimentation to be focused around the presumed MTD contour (corresponding to a TTL of 0.3), as shown in Figure 3.

**Table 1:** Prior mean and variances for model parameters used in the simulation study.

Model	Parameter	Mean	Variance
Six-parameter 2003	*α*_1_	0.43	0.11
	*β*_1_	7.65	5.71
	*α*_2_	0.43	0.08
	*β*_2_	7.80	3.99
	*α*_3_	1.00	0.90
	*β*_3_	1.00	0.90

Three-parameter 2009	*Δ*	1.00	0.50
	*ψ*	1.00	0.50
	*γ*	1.00	0.50

The prior mean probability of DLT, when each drug is used in isolation, is calculated from the model and used as the skeleton in the three-parameter model (the fixed *p*s and *q*s in Equation (2)). All parameters in the three-parameter model are given independent gamma(2,2) priors (mean 1 and variance 0.5). Note that Yin and Yuan 2009 also used these priors for *δ* and *ψ*, whereas they used a more vague gamma(0.1,0.1) prior for the interaction parameter *γ*. However, we found such a prior to be numerically unstable, particularly when calculating the information matrix, because sampling values of *γ* were very close to zero.

The first cohort is treated at the lowest dose combination (i.e. (0.2,0.2)), and two individuals are treated in each cohort. Escalation then proceeds using either decision rule *D*1 or *D*2, under a certain admissible design set ( Ω_1_, Ω_2_, or Ω_3_). The TTL is set to 30% and *ϵ* = 0.025; that is, for design *D*2, we further restrict our admissible set to dose combinations whose posterior mean probability is within the range (0.275,0.325). A total of 20 cohorts (40 patients) are treated in the trial. The final RPII dose combinations are selected as those that have been experimented on during the course of the Phase I trial and whose posterior mean probability of DLT is within *ϵ* of the TTL, that is the set

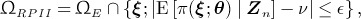
(6)

where Ω_*E*_ is the set of dose combinations experimented on during the trial. One thousand simulations were performed, in which the toxicity outcome for each patient is drawn from a Bernoulli distribution with the true probability of toxicity depending on the current dose combination. All analyses were carried out in R linked to the MCMC package JAGS 2011, and the code is available from the authors upon request.

### 5.1 Scenarios

Four scenarios are investigated. The first takes the true probability of toxicity at each dose combination from the prior mean, as specified from the six-parameter model (Table(a)). Both models are therefore expected to perform well under this scenario. Four of the prespecified dose combinations are MTDs, and it is desirable for the designs to recommend as many of these doses for Phase II experimentation as possible. In the second scenario, the probability of a DLT is higher than the specified prior mean probabilities for high-dose combinations (Table(b)). In this scenario, there are five possible MTDs, each with a probability of DLT equal to 30%. However, a one level increase above any MTD for either drug results in a large jump in the probability of a DLT to 45%. Hence, in this scenario, the risk of overdosing is high. The scenario was not derived from a specific choice of model parameters using either the three-parameter or six-parameter model; rather, the aim is to assess the robustness of the escalation procedure to model any prior misspecification. The third scenario assumes an asymmetric dose-toxicity surface (Table(c)), where drug A is more toxic than B over the dose range. This could arise if the prior is misspecified for the single-agent MTD dose for drug A. There are four possible MTDs in this scenario. The fourth scenario investigated assumes the dose-toxicity surface is relatively flat, with the probability of DLT ranging from 0.19 to 0.415 over the dose combinations. Here, there are nine possible MTDs (doses whose toxicity is within *ϵ* of the TTL).

**Table 2:** True toxicity probabilities for the simulations, with maximum tolerated doses shown in bold (doses within 0.025 of the target toxicity level).

Drug B	Drug A						
	0.2	0.5	0.7	0.8	0.9	0.95
	a) Scenario 1: in agreement with prior						
0.95	0.23	0.27	**0.30**	0.36	0.44	0.49
0.90	0.18	0.21	0.26	**0.30**	0.39	0.44
0.80	0.11	0.14	0.18	0.23	**0.30**	0.36
0.70	0.06	0.09	0.14	0.18	0.26	**0.30**
0.50	0.03	0.05	0.09	0.13	0.21	0.27
0.20	0.02	0.03	0.06	0.10	0.18	0.23

	b) Scenario 2: toxic						
0.95	0.23	**0.30**	0.45	0.50	0.55	0.60
0.90	0.18	0.21	**0.30**	0.45	0.50	0.55
0.80	0.11	0.14	0.18	**0.30**	0.45	0.50
0.70	0.06	0.09	0.14	0.18	**0.30**	0.45
0.50	0.03	0.05	0.09	0.13	0.21	**0.30**
0.20	0.02	0.03	0.06	0.10	0.18	0.23

	b) Scenario 3: asymmetric toxic						
0.95	**0.30**	0.38	0.48	0.58	0.68	0.78
0.90	0.22	**0.30**	0.40	0.50	0.60	0.70
0.80	0.17	0.25	0.35	0.45	0.50	0.60
0.70	0.12	0.20	**0.30**	0.40	0.45	0.55
0.50	0.06	0.14	0.24	0.34	0.39	0.49
0.20	0.02	0.10	0.20	**0.30**	0.35	0.45

	b) Scenario 4: flat						
0.95	0.265	**0.295**	0.325	0.355	0.385	0.415
0.90	0.250	**0.280**	**0.310**	0.340	0.370	0.400
0.80	0.235	0.265	**0.295**	0.325	0.355	0.385
0.70	0.220	0.250	**0.280**	**0.310**	0.340	0.370
0.50	0.205	0.235	0.265	**0.295**	0.325	0.355
0.20	0.190	0.220	0.250	**0.280**	**0.310**	0.340

### 5.2 Escalation: six-parameter model

**Figure 4:**
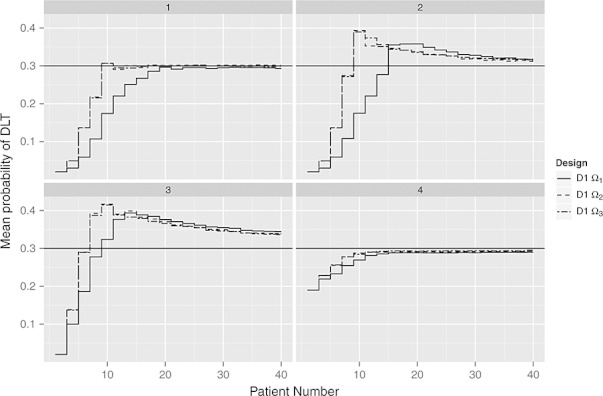
Mean probability of dose-limiting toxicity (DLT) for each individual recruited in the trial under Scenarios 1–4, for admissible sets Ω_1_ (neighbouring, excluding diagonal), Ω_2_ (neighbouring, including diagonal), and Ω_3_ (neighbouring, including diagonal and previously experimented dose combinations). The six-parameter model and decision rule *D*1 is used for all designs.

The mean probability of the DLT for each patient recruited in the trial is shown in Figure 4 for each scenario under the various admissible sets, using the six-parameter model and decision rule *D*1. A very similar escalation pattern is seen under the decision rule *D*2, and hence, these results are not plotted. In every scenario, designs using either the admissible set Ω_2_ or Ω_3_ escalate faster compared to those using Ω_1_. Under scenario 1, dosing stabilises around the TTL, on average, after 10 patients have been recruited. In contrast, the admissible set Ω_1_, which does not allow diagonal escalation, takes an average of 20 patients until the TTL is reached. This results in more patients receiving suboptimal doses. Scenarios 2 and 3 result in some overdosing, due to the unexpected toxic nature of the drug combination, before the designs start to converge down to the TTL. Designs Ω_2_ and Ω_3_ peak faster and at a higher toxicity level than the more conservative Ω_1_ design. Under the flat dose-toxicity surface of Scenario 4, all designs escalate at roughly the same rate and converge on the TTL from below.

### 5.3 Experimentation: six-parameter model

**Figure 5:**
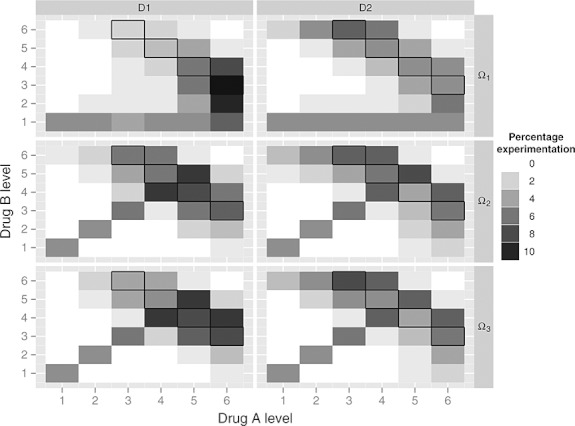
Percentage of experimentation at each dose combination under Scenario 1, for designs with decision rule *D*1 and *D*2, and admissible dose sets Ω_1_, Ω_2_, and Ω_3_. An outline is drawn around the four maximum tolerated dose (MTD) combinations. The six-parameter model is used for all designs.

Figure 5 illustrates the percentage of times each dose combination is experimented on under Scenario 1 for the competing designs. Using the admissible set Ω_1_, escalation generally proceeds along the margin of the two-dimensional space (i.e. drug B remains at its first level) before escalation to the TTL. This occurs because the prior for *β*_2_ is higher than *β*_1_. Hence, although no DLTs have been observed, at each decision point, an increase in drug A is estimated to get closer to the TTL than an increase in drug B. If the prior mean for *β*_2_ was less than that of *β*_1_ then, on average, drug B dose levels will be escalated first. In fact, only if the presumed dose-toxicity curve is convex (this happens if *β*_1_ < 1 and *β*_2_ < 1) does the design behave in a step-like fashion by escalating drug A and B in turns. Under the non-diagonal design (*D*1 Ω_1_), a large proportion of patients are treated at just one of the four MTDs, (*x*_*A*6_,*x*_*B*3_), whereas other MTDs are very rarely experimented on. Using the admissible set Ω_2_, escalation proceeds up the diagonal of the two-dimensional space, and experimentation among the four MTDs is more equally spread. The D-optimal designs all produce slightly more varied experimentations compared with designs *D*1, and this is especially true when the admissible dose set includes previously experimented doses ( Ω_3_).

Table shows how each design performs in terms of underdosing and overdosing patients recruited to the trial, under the varying scenarios. It is clear that operating characteristics are strongly dependent on the scenario investigated. Under Scenario 1, approximately 40% of all trial participants are treated at a target level (25%–34% toxicity). Designs that use a diagonal escalation strategy ( Ω_2_ and Ω_3_) tend to dose more patients at the target level under Scenarios 1-3, whereas very similar dosing profiles are seen in Scenario 4. However, these designs also tend to overdose more often compared with the more conservative Ω_1_ escalation. The D-optimal design (*D*2) slightly improves operating characteristics under the Ω_1_ strategy but does not consistently improve experimentation percentages under designs Ω_2_ and Ω_3_.

**Table 3:** Experimentation percentages for the six-parameter model under the various designs. The target toxicity range here is defined as 25%–34%.

Toxicity (%)	Design						
	*D*1 Ω_1_	*D*1 Ω_2_	*D*1 Ω_3_	*D*2 Ω_1_	*D*2 Ω_2_	*D*2 Ω_3_
Scenario 1: in agreement with prior						
Severe underdosing 0–14	22.3	17.4	17.9	21.7	17.1	17.0
Underdosing 15–24	22.4	16.6	16.5	21.3	19.1	19.0
Target dosing 25–34	39.3	42.1	40.7	40.4	39.9	40.5
Overdosing 35–44	15.9	23.7	24.7	16.4	23.6	23.3
Severe overdosing ≥ 45	0.1	0.3	0.2	0.2	0.3	0.2

Scenario 2: toxic						
Severe underdosing 0–14	22.1	18.4	18.6	22.2	17.3	17.7
Underdosing 15–24	24.5	16.6	14.8	23.2	19.1	17.8
Target dosing 25–34	29.7	36.5	37.8	29.6	35.9	36.8
Overdosing 35–44	18.3	20.8	20.6	19.4	19.9	20.1
Severe overdosing ≥ 45	5.3	7.6	8.1	5.4	7.8	7.7

Scenario 3: asymmetric toxic						
Severe underdosing 0–14	12.8	12.0	12.2	13.3	11.7	11.8
Underdosing 15–24	13.0	8.8	8.8	15.5	9.1	8.7
Target dosing 25–34	21.5	27.6	28.8	25.9	27.9	28.5
Overdosing 35–44	44.5	41.6	40.8	36.9	41.3	39.8
Severe overdosing ≥ 45	8.2	10.0	9.4	8.4	10.0	11.2

Scenario 4: flat						
Severe underdosing 0–14	0.0	0.0	0.0	0.0	0.0	0.0
Underdosing 15–24	31.6	28.5	29.5	32.5	26.5	28.7
Target dosing 25–34	59.3	59.1	56.2	60.5	61.8	58.9
Overdosing 35–44	9.2	12.5	14.4	7.0	11.6	12.4
Severe overdosing ≥ 45	0.0	0.0	0.0	0.0	0.0	0.0

### 5.4 Recommendation: six-parameter model

Table shows the toxicity of the RPII doses at the end of the trial. As to be expected, at the target level, the recommendation percentages are higher than the experimentation percentages shown in Table. It is interesting to note, however, that these percentages are still relatively low, suggesting that a much larger trial is required to have adequate power to recommend Phase II doses close to the target level. The advantage of the D-optimal designs (*D*2) is more pronounced when studying the recommendation percentages. RPII doses are generally less likely to be above the TTL, whereas higher percentages of the true MTDs are finally selected for Phase II compared with the *D*1 designs.

**Table 4:** Recommendation percentages for Phase II experimentation under the six-parameter model and the average percentage of true MTDs selected by the design.

Toxicity (%)	Design						
	*D*1 Ω_1_	*D*1 Ω_2_	*D*1 Ω_3_	*D*2 Ω_1_	*D*2 Ω_2_	*D*2 Ω_3_
Scenario 1: in agreement with prior						
Far below TTL 0–14	1.4	0.7	0.5	0.7	1.0	0.5
Below TTL 15–24	19.6	17.4	16.0	21.0	19.9	20.1
TTL 25–34	58.4	58.5	61.4	60.0	58.3	60.2
Above TTL 35–44	20.7	23.2	22.1	18.2	20.8	19.2
Far above TTL ≥ 45	0.0	0.2	0.1	0.1	0.1	0.0
% of MTDs selected	16	18	17	22	21	23

Scenario 2: toxic						
Far below TTL 0 - 14	1.0	1.0	0.6	1.3	1.6	1.4
Below TTL 15–24	25.1	19.6	21.8	24.5	23.2	24.6
TTL 25–34	47.2	55.5	51.8	49.6	51.9	53.1
Above TTL 35–44	23.5	21.9	23.3	21.7	21.1	19.0
Far above TTL ≥ 45	3.3	2.0	2.5	2.8	2.2	1.9
% of MTDs selected	18	21	18	27	24	25

Scenario 3: asymmetric toxic						
Far below TTL 0–14	1.4	0.8	0.8	1.2	1.2	0.7
Below TTL 15–24	10.1	10.8	10.3	10.8	11.1	10.9
TTL 25–34	29.6	34.9	36.9	30.9	33.6	34.9
Above TTL 35–44	53.9	49.8	47.6	50.2	49.2	48.1
Far above TTL ≥ 45	4.9	3.7	4.5	6.9	4.8	5.5
% of MTDs selected	8	10	9	12	11	13

Scenario 4: flat						
Far below TTL 0–14	0.0	0.0	0.0	0.0	0.0	0.0
Below TTL 15–24	13.7	12.3	11.9	14.0	11.7	11.6
TTL 25–34	73.1	75.4	70.8	76.2	75.7	74.8
Above TTL 35–44	13.1	12.3	17.3	9.8	12.6	13.6
Far above TTL ≥ 45	0.0	0.0	0.0	0.0	0.0	0.0
% of MTDs selected	7	7	6	9	8	8

TTL, target toxicity level. MTD, maximum tolerated dose.

### 5.5 Recommendation: three-parameter model

It should be noted that a formal comparison between the operating characteristics of the three-parameter and six-parameter models is difficult because of the choice of prior distributions. The priors chosen for the two models in this analysis were not intended to give matching dose-toxicity surfaces, in terms of either prior means or variances. Therefore, in this paper, we have adopted a pragmatic approach, using priors that have been previously recommended.

Table shows the toxicity of the RPII doses using the three-parameter model. A similar pattern is observed, as seen with the investigations using the six-parameter model, with reasonable operating characteristics under Scenarios 1 and 4, whereas a fair proportion of RPII doses under Scenarios 2 and 3 are above the true TTL. In contrast to the six-parameter model, the *D*2 designs appear to reduce the proportion of RPII doses that have toxicity at the TTL. However, the percentage of true MTDs recommended under these designs does increase. This apparent contradiction arises because the D-optimal designs are recommending more Phase II doses, both correctly at the TTL and incorrectly outside of the TTL. The diagonal designs ( Ω_2_ and Ω_3_) generally recommend a higher proportion of RPII doses at the TTL, but at the expense of increased overdosing of patients during the trial (experimentation results for three-parameter model not shown).

**Table 5 tbl5:** Recommendation percentages for Phase II experimentation under the three-parameter model and the average percentage of true MTDs selected by the design.

Toxicity (%)	Design						
	*D*1 Ω_1_	*D*1 Ω_2_	*D*1 Ω_3_	*D*2 Ω_1_	*D*2 Ω_2_	*D*2 Ω_3_
Scenario 1: in agreement with prior						
Far below TTL 0–14	1.6	2.3	2.0	3.2	3.5	3.5
Below TTL 15–24	26.0	24.1	21.8	27.2	25.4	25.8
TTL 25–34	49.7	47.3	50.1	47.3	43.9	43.7
Above TTL 35–44	21.7	25.3	24.9	21.5	26.5	26.1
Far above TTL ≥ 45	1.0	1.1	1.1	0.8	0.7	0.8
% of MTDs selected	14	14	15	19	15	14

Scenario 2: toxic						
Far below TTL 0–14	3.5	2.7	2.6	4.8	5.1	4.9
Below TTL 15–24	31.5	24.9	25.2	32.8	29.2	29.4
TTL 25–34	39.4	46.7	47.9	34.7	40.3	40.9
Above TTL 35 - 44	20.3	21.0	20.8	21.6	20.9	19.2
Far above TTL ≥ 45	5.3	4.7	3.5	6.2	4.5	5.6
% of MTDs selected	17	20	19	21	20	19

Scenario 3: asymmetric toxic						
Far below TTL 0–14	1.2	1.4	1.5	1.3	2.0	1.5
Below TTL 15–24	17.4	18.7	19.0	18.7	18.3	17.6
TTL 25–34	39.0	39.2	39.1	35.8	39.1	40.5
Above TTL 35–44	38.1	37.6	36.9	39.0	36.1	35.9
Far above TTL ≥ 45	4.3	3.0	3.6	5.2	4.4	4.5
% of MTDs selected	11	11	10	15	13	13

Scenario 4: flat						
Far below TTL 0–14	0.0	0.0	0.0	0.0	0.0	0.0
Below TTL 15–24	17.1	11.7	11.1	16.3	12.4	11.0
TTL 25–34	71.5	74.5	73.7	69.0	73.1	72.9
Above TTL 35–44	11.4	13.8	15.1	14.7	14.5	16.1
Far above TTL ≥ 45	0.0	0.0	0.0	0.0	0.0	0.0
% of MTDs selected	6	5	5	6	7	7

TTL, target toxicity level. MTD, maximum tolerated dose.

Under these prior specifications, the six-parameter model tends to outperform the three-parameter model in Scenarios 1, 2 and 4 in terms of RPII doses at the TTL and the overall percentage of the true selected MTDs (Tables and 5). Interestingly, however, the three-parameter model appears to perform much better under Scenario 3 when the dose-toxicity curve is asymmetric.

## 6 DISCUSSION

In this paper, we have shown a diagonal escalation strategy to be more efficient in that it reaches the TTL quicker, with approximately 20%–50% fewer patients dosed at suboptimal levels and correctly recommends more MTDs at the end of the trial. We found that the non-diagonal strategies start by escalating a single agent while keeping the other agent fixed, and we did not see the step-like escalation that was anticipated. This behaviour may be undesirable because the aim may be to show safety when both drugs are being used in reasonable quantity. For example, in our simulation studies, drug A was first escalated near to its MTD, whereas drug B remained fixed at the lowest level. This results in some MTDs at the end of the trial being rarely recommended. The planned sample size of the trial must, therefore, take into account the proposed escalation strategy.

The tradeoff for using a diagonal escalation strategy is that it will lead to an increased risk of overdosing within some trial patients; the extent of which depends on the underlying dose-toxicity surface and the proposed increments in dose for each drug. The severity of this consequence will depend very much on the disease and the drugs under consideration. However, if escalation to potentially overly toxic doses is of major concern, then the dose range should be subdivided into a finer grid, if possible. In other words, the prespecification of the doses should take into account whether a diagonal or non-diagonal strategy is to be used in the escalation procedure. The flexibility of model-based adaptive designs present an advantage here because dose levels may also be refined during the course of the trial. The models would treat the added dose combinations as additional design points for consideration when deciding the next cohort's dose, with the admissible dose set also being updated appropriately. Researchers must, therefore, consider these design issues carefully in order to ensure that overdosing is not too severe and is kept to a minimum.

Designs based on Bayesian D-optimality criteria are shown to allow better traversing of the dose space. This, in turn, slightly improves the percentage of correctly recommended MTDs. When combined with a strategy that allows diagonal escalation

and experimentation at previous administered doses, the performance of the D-optimal design is further enhanced. Such escalation strategies are therefore important, especially for Phase I trials with a large number of prespecified dose combinations.

In three out of the four scenarios investigated, the six-parameter model has been found to outperform the three-parameter model when the objective is to identify more than one MTD. However, care must be taken when comparing between models because of the different prior specifications. One advantage of the CRM approach in the single-agent trials has been that underparameterised models can be used to provide good local fit at the TTL. However, in combination therapy trials, models need to be more flexible, and hence complex, in order to provide a good approximation of the whole dose-toxicity contour at the TTL. In our experience, we have found that the six-parameter model allows a more explicit specification of prior information from single-agent data, with four of the six parameters specifically related to this. Prior information can be elicited from experts, as was the case of the Gemcitabine and Cyclophosphamide trial 2003, or priors can be obtained directly from previous Phase I trial data. For the latter, some discounting (i.e. increasing the prior variance) may be necessary if previous trials are conducted in different populations and with different protocols.
